# Dimethyl 2,6-dimethyl-4-{3-[4-(methyl­sulfan­yl)phen­yl]-1*H*-pyrazol-4-yl}-1,4-dihydro­pyridine-3,5-dicarboxyl­ate monohydrate

**DOI:** 10.1107/S1600536812045333

**Published:** 2012-11-10

**Authors:** Arun M. Islor, A. M. Vijesh, Thomas Gerber, Eric Hosten, Richard Betz

**Affiliations:** a National Institute of Technology-Karnataka, Department of Chemistry, Organic Chemistry Laboratory, Surathkal, Mangalore 575 025, India; b GITAM University, Department of Engineering Chemistry, GIT, Rushikonda, Visakhapatnam, A.P. 530 045, India; c Nelson Mandela Metropolitan University, Summerstrand Campus, Department of Chemistry, University Way, Summerstrand, PO Box 77000, Port Elizabeth, 6031, South Africa

## Abstract

In the title compound, C_21_H_23_N_3_O_4_S·H_2_O, the methyl­sulfanyl group is disordered over two sets of sites with site-occupancy factors of 0.631 (11) and 0.369 (11). The dihydro­pyridine ring adopts an *E*_4_ conformation. In the crystal, classical O—H⋯N, O—H⋯O and N—H⋯O hydrogen bonds, as well as C—H⋯O and C—H⋯S contacts, connect the mol­ecules into a three-dimensional network.

## Related literature

For general information about the pharmacological importance of 1,4-dihydro­pyridine-based drugs, see: Janis & Triggle (1983 ▶); Boecker & Guengerich (1986 ▶); Gordeev *et al.* (1996 ▶); Buhler & Kiowski (1987 ▶); Vo *et al.* (1995 ▶). For puckering analysis of cyclic motifs, see: Cremer & Pople (1975 ▶). For graph-set analysis of hydrogen bonds, see: Etter *et al.* (1990 ▶); Bernstein *et al.* (1995 ▶).
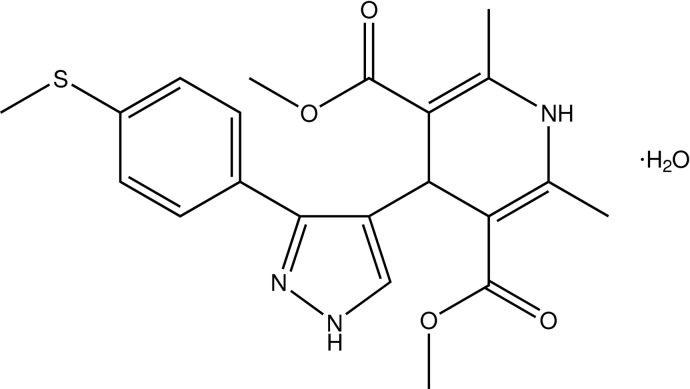


## Experimental

### 

#### Crystal data

C_21_H_23_N_3_O_4_S·H_2_O
*M* *_r_* = 431.50
Monoclinic, 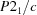

*a* = 10.5542 (2) Å
*b* = 14.7260 (2) Å
*c* = 14.5377 (2) Å
β = 110.106 (1)°
*V* = 2121.77 (6) Å^3^
*Z* = 4
Mo *K*α radiation
μ = 0.19 mm^−1^
*T* = 200 K
0.27 × 0.23 × 0.20 mm

#### Data collection

Bruker APEXII CCD diffractometer
Absorption correction: multi-scan (*SADABS*; Bruker, 2008 ▶) *T*_min_ = 0.950, *T*_max_ = 0.963
20236 measured reflections
5267 independent reflections
4311 reflections with *I* > 2σ(*I*)
*R*_int_ = 0.019

#### Refinement

*R*[*F* ^2^ > 2σ(*F* ^2^)] = 0.040
*wR*(*F* ^2^) = 0.111
*S* = 1.03
5267 reflections
312 parameters
H atoms treated by a mixture of independent and constrained refinement
Δρ_max_ = 0.30 e Å^−3^
Δρ_min_ = −0.27 e Å^−3^

### 

Data collection: *APEX2* (Bruker, 2010 ▶); cell refinement: *SAINT* (Bruker, 2010 ▶); data reduction: *SAINT*; program(s) used to solve structure: *SHELXS97* (Sheldrick, 2008 ▶); program(s) used to refine structure: *SHELXL97* (Sheldrick, 2008 ▶); molecular graphics: *ORTEP-3* (Farrugia, 1997 ▶) and *Mercury* (Macrae *et al.*, 2008 ▶); software used to prepare material for publication: *SHELXL97* and *PLATON* (Spek, 2009 ▶).

## Supplementary Material

Crystal structure: contains datablock(s) I, global. DOI: 10.1107/S1600536812045333/rn2110sup1.cif

Supplementary material file. DOI: 10.1107/S1600536812045333/rn2110Isup2.cdx

Structure factors: contains datablock(s) I. DOI: 10.1107/S1600536812045333/rn2110Isup3.hkl

Supplementary material file. DOI: 10.1107/S1600536812045333/rn2110Isup4.cml

Additional supplementary materials:  crystallographic information; 3D view; checkCIF report

## Figures and Tables

**Table 1:** Hydrogen-bond geometry (Å, °)

*D*—H⋯*A*	*D*—H	H⋯*A*	*D*⋯*A*	*D*—H⋯*A*
O8—H8*A*⋯N22^i^	0.83 (3)	2.09 (3)	2.8982 (18)	167 (2)
O8—H8*B*⋯O4^ii^	0.84 (3)	2.09 (3)	2.8989 (19)	164 (2)
N21—H21⋯O2^iii^	0.884 (19)	1.985 (19)	2.8505 (15)	165.9 (17)
N31—H31*A*⋯O8^iv^	0.908 (19)	1.965 (19)	2.8561 (18)	166.6 (17)
C23—H23⋯S1*A* ^v^	0.95	2.79	3.637 (3)	149

Symmetry codes: (i) 
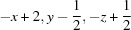
; (ii) 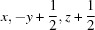; (iii) 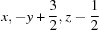; (iv) 
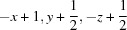
; (v) 
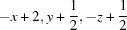
.

**Fig. 1.**
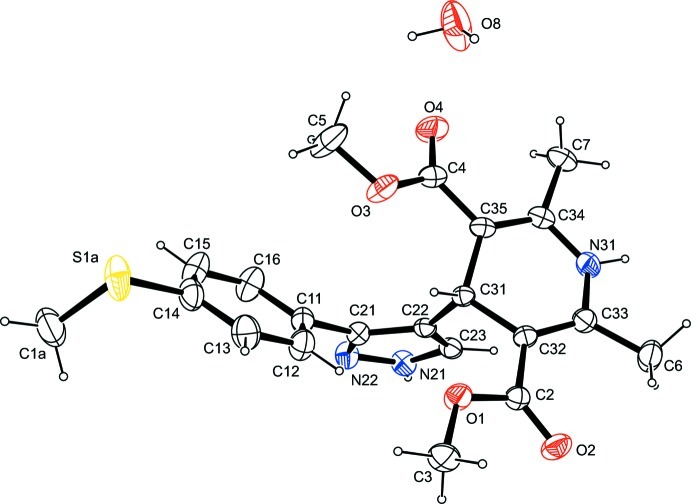
The molecular structure of the title compound, with atom labels and anisotropic displacement ellipsoids (drawn at 50% probability level). For clarity, only the major component of the split model is depicted.

**Fig. 2.**
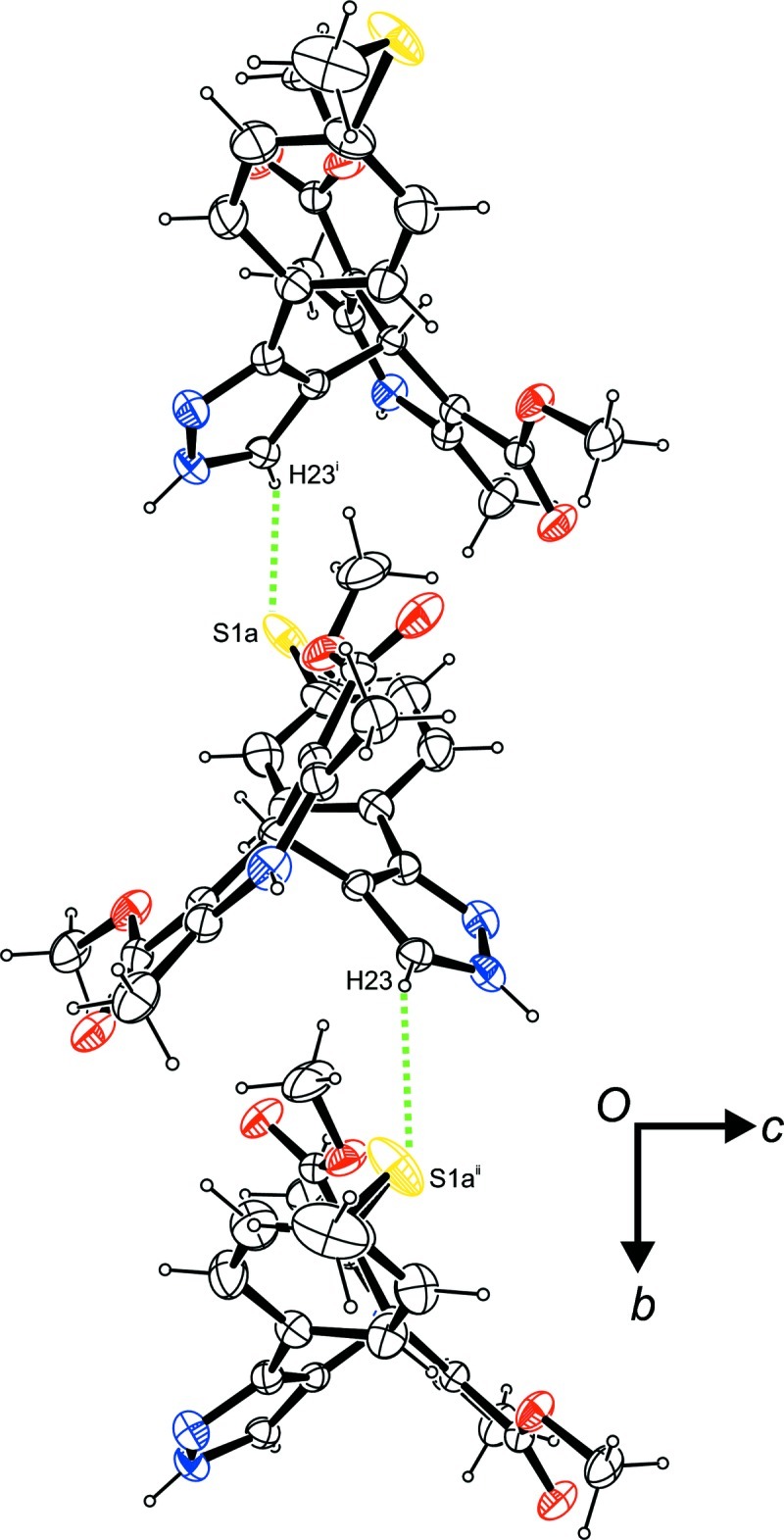
Intermolecular contacts, viewed along [-1 0 0]. For clarity, only the major component of the split model and only the C–H···S contacts necessitating a C11(9) descriptor are depicted. Symmetry operators: i 2 - x,-1/2 + y,1/2 - z; ii 2 - x,1/2 + y,1/2 - z.

## supplementary crystallographic information

### Comment

In recent years, considerable attention has been paid to the synthesis of 1,4-dihydropyridines owing to their significant biological activity. 1,4-Dihydropyridine-containing drugs (1,4-DHPs), such as nifedipine, nicardipine, amlodipine, felodipine and others have been found to be useful as calcium channel blockers (Janis & Triggle, 1983; Boecker & Guengerich, 1986; Gordeev *et al.*, 1996) and are used most frequently as cardiovascular agents for the treatment of hypertension (Buhler & Kiowski, 1987). A number of DHP derivatives are employed as potential drug candidates for the treatment of congestive heart failure (Vo *et al.*, 1995). In continuation of our ongoing interest in pharmaceutically active compounds, the title compound was synthesized to study its crystal structure.

The compound is the hydrate of a mixed pyrazole-1,4-dihydropyridine compound. The thiomethyl group is disordered over two positions with site occupancy factors of 0.631 (11) and 0.369 (11). According to a puckering analysis (Cremer & Pople, 1975), the dihydropyridine ring adopts an *E*_4_ conformation with the flap atom on C31 (*E*_C31_). The least-squares planes defined by the carbon atoms of the phenyl group and the intracyclic atoms of the pyrazole ring enclose an angle of 48.42 (8) °. At the same time, the aforementioned planes intersect with the least-squares plane defined by the atoms of the 1,4-dihydropyridine ring at angles of 45.18 (7) ° and 86.12 (7) °, respectively.

In the crystal, classical hydrogen bonds of the O–H···N, O–H···O and N–H..O type can be observed that are supported by all nitrogen- and oxygen-bound hydrogen atoms. The bifurcated C H···O contact may influence the eclipsed ester substituent conformation with respect to this group. Furthermore, an intermolecular C–H···S contact is present falling short by more than 0.2 Å of the sum of van-der-Waals radii of the corresponding atoms. These contacts connect the entities in the crystal structure to a three-dimensional network. In terms of graph-set analysis (Etter *et al.*, 1990; Bernstein *et al.*, 1995), the descriptor for these contacts is *S*(5)*S*(5)*DDDC*^1^_1_(8)*C*^1^_1_(9) on the unary level. The *C*^1^_1_(9) descriptor detailing the intermolecular C–H···S contacts is shown in Figure 2. Metrical parameters as well as information about the symmetry of these contacts are summarized in Table 1. The shortest intercentroid distance between two aromatic systems was measured at 5.2965 (8) Å and is apparent between the pyrazole and the phenyl moiety in two neighbouring molecules.

### Experimental

3-(4-methylsulfanyl-phenyl)-1*H*-pyrazole-4-carbaldehyde (0.2 g, 0.9 mmol), methylacetoacetate (0.21 g, 1.8 mmol) and ammonium acetate (0.07 g, 0.9 mmol) in methanol (20 mL) were heated under reflux in an oil bath for 8 h. After completion of the reaction, the reaction mixture was concentrated and poured onto crushed ice. The precipitate was filtered and washed with water. The resulting solid was recrystallized from hot methanol, yield: 0.32 g (84%).

### Refinement

Carbon-bound H atoms were placed in calculated positions (C—H 0.95 Å for aromatic carbon atoms and C—H 1.00 Å for the methine group) and were included in the refinement in the riding model approximation, with *U*(H) set to 1.2*U*_eq_(C). The H atoms of the methyl groups were allowed to rotate with a fixed angle around the C—C bond to best fit the experimental electron density (HFIX 137 in the *SHELX* program suite (Sheldrick, 2008), with *U*(H) set to 1.5*U*_eq_(C). All nitrogen- and oxygen-bound H atoms were located on a difference Fourier map and refined freely.

### Crystal data

**Table:**

C_21_H_23_N_3_O_4_S·H_2_O	*F*(000) = 912
*M**_r_* = 431.50	*D*_x_ = 1.351 Mg m^−^^3^
Monoclinic, *P*2_1_/*c*	Melting point = 467–469 K
Hall symbol: -P 2ybc	Mo *K*α radiation, λ = 0.71073 Å
*a* = 10.5542 (2) Å	Cell parameters from 9343 reflections
*b* = 14.7260 (2) Å	θ = 2.5–28.2°
*c* = 14.5377 (2) Å	µ = 0.19 mm^−^^1^
β = 110.106 (1)°	*T* = 200 K
*V* = 2121.77 (6) Å^3^	Block, colourless
*Z* = 4	0.27 × 0.23 × 0.20 mm

### Data collection

**Table:**

Bruker APEXII CCD diffractometer	5267 independent reflections
Radiation source: fine-focus sealed tube	4311 reflections with *I* > 2σ(*I*)
Graphite monochromator	*R*_int_ = 0.019
φ and ω scans	θ_max_ = 28.3°, θ_min_ = 2.1°
Absorption correction: multi-scan (*SADABS*; Bruker, 2008)	*h* = −14→13
*T*_min_ = 0.950, *T*_max_ = 0.963	*k* = −14→19
20236 measured reflections	*l* = −13→19

### Refinement

**Table:**

Refinement on *F*^2^	Primary atom site location: structure-invariant direct methods
Least-squares matrix: full	Secondary atom site location: difference Fourier map
*R*[*F*^2^ > 2σ(*F*^2^)] = 0.040	Hydrogen site location: inferred from neighbouring sites
*wR*(*F*^2^) = 0.111	H atoms treated by a mixture of independent and constrained refinement
*S* = 1.03	*w* = 1/[σ^2^(*F*_o_^2^) + (0.056*P*)^2^ + 0.7436*P*] where *P* = (*F*_o_^2^ + 2*F*_c_^2^)/3
5267 reflections	(Δ/σ)_max_ = 0.001
312 parameters	Δρ_max_ = 0.30 e Å^−^^3^
0 restraints	Δρ_min_ = −0.27 e Å^−^^3^

### Fractional atomic coordinates and isotropic or equivalent isotropic displacement parameters (Å^2^)

**Table:**

	*x*	*y*	*z*	*U*_iso_*/*U*_eq_	Occ. (<1)
O1	0.93564 (10)	0.59820 (7)	0.43428 (7)	0.0310 (2)	
O2	0.78574 (10)	0.70794 (7)	0.42595 (7)	0.0347 (2)	
O3	0.87427 (11)	0.35098 (7)	0.22830 (8)	0.0376 (3)	
O4	0.69528 (12)	0.32275 (8)	0.09516 (8)	0.0422 (3)	
O8	0.73981 (13)	0.12824 (12)	0.41554 (11)	0.0621 (4)	
H8A	0.820 (3)	0.1239 (17)	0.4195 (18)	0.073 (7)*	
H8B	0.743 (3)	0.1385 (17)	0.473 (2)	0.075 (8)*	
N21	0.88151 (11)	0.65657 (8)	0.07373 (8)	0.0278 (2)	
H21	0.8649 (18)	0.6971 (13)	0.0262 (13)	0.041 (5)*	
N22	0.99846 (12)	0.60935 (8)	0.10597 (8)	0.0294 (3)	
N31	0.51897 (12)	0.55198 (8)	0.18783 (9)	0.0286 (2)	
H31A	0.431 (2)	0.5681 (12)	0.1579 (13)	0.043 (5)*	
C2	0.81451 (13)	0.63803 (8)	0.39268 (9)	0.0231 (2)	
C3	1.02617 (15)	0.64046 (11)	0.52148 (10)	0.0346 (3)	
H3A	0.9833	0.6430	0.5715	0.052*	
H3B	1.0472	0.7022	0.5061	0.052*	
H3C	1.1096	0.6050	0.5463	0.052*	
C4	0.75074 (14)	0.37067 (9)	0.16581 (10)	0.0270 (3)	
C5	0.9362 (2)	0.27053 (11)	0.20646 (14)	0.0543 (5)	
H5A	1.0260	0.2627	0.2559	0.081*	
H5B	0.9444	0.2766	0.1416	0.081*	
H5C	0.8803	0.2175	0.2070	0.081*	
C6	0.51172 (15)	0.66544 (11)	0.30489 (12)	0.0373 (3)	
H6A	0.5579	0.6767	0.3749	0.056*	
H6B	0.4234	0.6380	0.2948	0.056*	
H6C	0.4995	0.7230	0.2690	0.056*	
C7	0.45802 (15)	0.42238 (10)	0.07938 (11)	0.0347 (3)	
H7A	0.4535	0.3609	0.1038	0.052*	
H7B	0.4796	0.4191	0.0191	0.052*	
H7C	0.3707	0.4526	0.0658	0.052*	
C11	1.10148 (13)	0.49866 (9)	0.23494 (10)	0.0267 (3)	
C12	1.14884 (14)	0.49982 (10)	0.33673 (11)	0.0336 (3)	
H12	1.1077	0.5389	0.3702	0.040*	
C13	1.25535 (15)	0.44469 (11)	0.39020 (12)	0.0384 (3)	
H13	1.2866	0.4466	0.4597	0.046*	
C14	1.31631 (14)	0.38692 (10)	0.34276 (12)	0.0360 (3)	
C15	1.26844 (18)	0.38458 (12)	0.24128 (13)	0.0446 (4)	
H15	1.3083	0.3444	0.2079	0.054*	
C16	1.16273 (17)	0.44037 (11)	0.18802 (12)	0.0398 (4)	
H16	1.1319	0.4386	0.1185	0.048*	
C21	0.98781 (13)	0.55804 (8)	0.17910 (9)	0.0238 (3)	
C22	0.86336 (12)	0.57278 (8)	0.19351 (9)	0.0205 (2)	
C23	0.79950 (13)	0.63695 (8)	0.12396 (9)	0.0239 (3)	
H23	0.7131	0.6626	0.1135	0.029*	
C31	0.79937 (12)	0.52258 (8)	0.25760 (8)	0.0200 (2)	
H31	0.8713	0.4887	0.3095	0.024*	
C32	0.72985 (12)	0.58775 (8)	0.30658 (9)	0.0217 (2)	
C33	0.59493 (13)	0.60214 (9)	0.26803 (9)	0.0253 (3)	
C34	0.56572 (13)	0.47545 (9)	0.15537 (9)	0.0256 (3)	
C35	0.69821 (13)	0.45444 (8)	0.19347 (9)	0.0228 (2)	
S1A	1.4500 (2)	0.31690 (16)	0.41722 (17)	0.0465 (5)	0.631 (11)
C1A	1.5934 (3)	0.3614 (3)	0.3947 (5)	0.0589 (14)	0.631 (11)
H1A	1.6736	0.3261	0.4315	0.088*	0.631 (11)
H1B	1.6066	0.4250	0.4156	0.088*	0.631 (11)
H1C	1.5791	0.3575	0.3245	0.088*	0.631 (11)
S1B	1.4470 (4)	0.3094 (3)	0.4019 (4)	0.0648 (12)	0.369 (11)
C1B	1.5788 (7)	0.3823 (5)	0.4539 (10)	0.069 (3)	0.369 (11)
H1D	1.6579	0.3478	0.4939	0.104*	0.369 (11)
H1E	1.5536	0.4262	0.4953	0.104*	0.369 (11)
H1F	1.6002	0.4147	0.4022	0.104*	0.369 (11)

### Atomic displacement parameters (Å^2^)

**Table:**

	*U*^11^	*U*^22^	*U*^33^	*U*^12^	*U*^13^	*U*^23^
O1	0.0267 (5)	0.0338 (5)	0.0252 (5)	0.0035 (4)	−0.0004 (4)	−0.0091 (4)
O2	0.0364 (5)	0.0316 (5)	0.0311 (5)	0.0039 (4)	0.0051 (4)	−0.0119 (4)
O3	0.0451 (6)	0.0263 (5)	0.0333 (5)	0.0110 (4)	0.0031 (4)	−0.0057 (4)
O4	0.0466 (6)	0.0377 (6)	0.0384 (6)	−0.0048 (5)	0.0098 (5)	−0.0183 (5)
O8	0.0280 (6)	0.1059 (13)	0.0516 (8)	−0.0074 (7)	0.0129 (6)	−0.0297 (8)
N21	0.0281 (6)	0.0282 (6)	0.0250 (5)	0.0005 (4)	0.0065 (4)	0.0076 (5)
N22	0.0279 (6)	0.0324 (6)	0.0292 (6)	0.0028 (5)	0.0115 (5)	0.0055 (5)
N31	0.0204 (5)	0.0331 (6)	0.0287 (6)	−0.0013 (4)	0.0039 (4)	−0.0032 (5)
C2	0.0254 (6)	0.0240 (6)	0.0200 (6)	−0.0012 (5)	0.0080 (5)	−0.0011 (5)
C3	0.0311 (7)	0.0393 (8)	0.0249 (7)	−0.0018 (6)	−0.0013 (5)	−0.0074 (6)
C4	0.0353 (7)	0.0219 (6)	0.0248 (6)	−0.0045 (5)	0.0114 (5)	−0.0010 (5)
C5	0.0691 (12)	0.0330 (8)	0.0528 (10)	0.0237 (8)	0.0107 (9)	−0.0065 (8)
C6	0.0290 (7)	0.0438 (8)	0.0393 (8)	0.0073 (6)	0.0121 (6)	−0.0071 (7)
C7	0.0308 (7)	0.0377 (7)	0.0314 (7)	−0.0130 (6)	0.0052 (6)	−0.0059 (6)
C11	0.0242 (6)	0.0268 (6)	0.0304 (7)	0.0024 (5)	0.0113 (5)	0.0036 (5)
C12	0.0297 (7)	0.0388 (8)	0.0308 (7)	0.0075 (6)	0.0084 (6)	−0.0013 (6)
C13	0.0308 (7)	0.0469 (9)	0.0330 (8)	0.0058 (6)	0.0052 (6)	0.0053 (7)
C14	0.0257 (7)	0.0336 (7)	0.0481 (9)	0.0059 (6)	0.0121 (6)	0.0123 (6)
C15	0.0476 (9)	0.0418 (9)	0.0508 (10)	0.0198 (7)	0.0250 (8)	0.0076 (7)
C16	0.0461 (9)	0.0429 (8)	0.0336 (8)	0.0150 (7)	0.0180 (7)	0.0053 (7)
C21	0.0246 (6)	0.0237 (6)	0.0230 (6)	0.0007 (5)	0.0082 (5)	0.0002 (5)
C22	0.0210 (5)	0.0189 (5)	0.0198 (5)	−0.0022 (4)	0.0046 (4)	−0.0025 (4)
C23	0.0220 (6)	0.0225 (6)	0.0245 (6)	−0.0023 (5)	0.0045 (5)	−0.0003 (5)
C31	0.0212 (5)	0.0189 (5)	0.0185 (5)	−0.0008 (4)	0.0049 (4)	−0.0012 (4)
C32	0.0237 (6)	0.0213 (5)	0.0199 (6)	−0.0006 (4)	0.0070 (5)	−0.0016 (5)
C33	0.0260 (6)	0.0264 (6)	0.0240 (6)	−0.0009 (5)	0.0090 (5)	−0.0009 (5)
C34	0.0275 (6)	0.0256 (6)	0.0226 (6)	−0.0071 (5)	0.0071 (5)	−0.0006 (5)
C35	0.0278 (6)	0.0208 (6)	0.0195 (5)	−0.0053 (5)	0.0078 (5)	−0.0019 (5)
S1A	0.0298 (7)	0.0559 (11)	0.0562 (8)	0.0169 (7)	0.0179 (5)	0.0368 (7)
C1A	0.0275 (14)	0.051 (2)	0.094 (3)	−0.0008 (13)	0.0159 (17)	0.017 (2)
S1B	0.0287 (12)	0.0245 (10)	0.121 (3)	0.0001 (8)	−0.0002 (13)	−0.0005 (14)
C1B	0.033 (3)	0.053 (4)	0.101 (8)	−0.003 (2)	−0.003 (3)	0.013 (4)

### Geometric parameters (Å, º)

**Table:**

O1—C2	1.3464 (16)	C11—C16	1.387 (2)
O1—C3	1.4402 (16)	C11—C12	1.390 (2)
O2—C2	1.2193 (16)	C11—C21	1.4810 (17)
O3—C4	1.3397 (17)	C12—C13	1.388 (2)
O3—C5	1.4403 (18)	C12—H12	0.9500
O4—C4	1.2175 (16)	C13—C14	1.385 (2)
O8—H8A	0.83 (3)	C13—H13	0.9500
O8—H8B	0.84 (3)	C14—C15	1.386 (2)
N21—C23	1.3417 (17)	C14—S1B	1.770 (4)
N21—N22	1.3524 (16)	C14—S1A	1.782 (3)
N21—H21	0.884 (19)	C15—C16	1.388 (2)
N22—C21	1.3402 (17)	C15—H15	0.9500
N31—C34	1.3770 (18)	C16—H16	0.9500
N31—C33	1.3802 (17)	C21—C22	1.4161 (17)
N31—H31A	0.908 (19)	C22—C23	1.3788 (17)
C2—C32	1.4644 (17)	C22—C31	1.5177 (16)
C3—H3A	0.9800	C23—H23	0.9500
C3—H3B	0.9800	C31—C32	1.5250 (16)
C3—H3C	0.9800	C31—C35	1.5270 (16)
C4—C35	1.4642 (18)	C31—H31	1.0000
C5—H5A	0.9800	C32—C33	1.3557 (18)
C5—H5B	0.9800	C34—C35	1.3511 (18)
C5—H5C	0.9800	S1A—C1A	1.780 (4)
C6—C33	1.5004 (19)	C1A—H1A	0.9800
C6—H6A	0.9800	C1A—H1B	0.9800
C6—H6B	0.9800	C1A—H1C	0.9800
C6—H6C	0.9800	S1B—C1B	1.713 (8)
C7—C34	1.5034 (17)	C1B—H1D	0.9800
C7—H7A	0.9800	C1B—H1E	0.9800
C7—H7B	0.9800	C1B—H1F	0.9800
C7—H7C	0.9800		
			
C2—O1—C3	116.57 (10)	C12—C13—H13	119.8
C4—O3—C5	115.99 (12)	C13—C14—C15	118.92 (13)
H8A—O8—H8B	105 (2)	C13—C14—S1B	124.9 (2)
C23—N21—N22	112.54 (11)	C15—C14—S1B	116.1 (2)
C23—N21—H21	125.4 (12)	C13—C14—S1A	117.34 (15)
N22—N21—H21	122.0 (12)	C15—C14—S1A	123.72 (15)
C21—N22—N21	104.44 (11)	C14—C15—C16	120.55 (15)
C34—N31—C33	123.70 (11)	C14—C15—H15	119.7
C34—N31—H31A	118.4 (12)	C16—C15—H15	119.7
C33—N31—H31A	117.7 (12)	C11—C16—C15	120.89 (15)
O2—C2—O1	121.14 (11)	C11—C16—H16	119.6
O2—C2—C32	127.16 (12)	C15—C16—H16	119.6
O1—C2—C32	111.70 (10)	N22—C21—C22	111.46 (11)
O1—C3—H3A	109.5	N22—C21—C11	119.65 (11)
O1—C3—H3B	109.5	C22—C21—C11	128.85 (11)
H3A—C3—H3B	109.5	C23—C22—C21	103.95 (11)
O1—C3—H3C	109.5	C23—C22—C31	125.14 (11)
H3A—C3—H3C	109.5	C21—C22—C31	130.33 (11)
H3B—C3—H3C	109.5	N21—C23—C22	107.61 (11)
O4—C4—O3	121.34 (13)	N21—C23—H23	126.2
O4—C4—C35	127.04 (13)	C22—C23—H23	126.2
O3—C4—C35	111.61 (11)	C22—C31—C32	111.54 (10)
O3—C5—H5A	109.5	C22—C31—C35	108.10 (9)
O3—C5—H5B	109.5	C32—C31—C35	110.55 (10)
H5A—C5—H5B	109.5	C22—C31—H31	108.9
O3—C5—H5C	109.5	C32—C31—H31	108.9
H5A—C5—H5C	109.5	C35—C31—H31	108.9
H5B—C5—H5C	109.5	C33—C32—C2	121.32 (11)
C33—C6—H6A	109.5	C33—C32—C31	120.73 (11)
C33—C6—H6B	109.5	C2—C32—C31	117.86 (10)
H6A—C6—H6B	109.5	C32—C33—N31	119.22 (12)
C33—C6—H6C	109.5	C32—C33—C6	127.59 (12)
H6A—C6—H6C	109.5	N31—C33—C6	113.13 (12)
H6B—C6—H6C	109.5	C35—C34—N31	119.29 (11)
C34—C7—H7A	109.5	C35—C34—C7	126.64 (12)
C34—C7—H7B	109.5	N31—C34—C7	114.07 (12)
H7A—C7—H7B	109.5	C34—C35—C4	121.10 (11)
C34—C7—H7C	109.5	C34—C35—C31	120.51 (11)
H7A—C7—H7C	109.5	C4—C35—C31	118.04 (11)
H7B—C7—H7C	109.5	C1A—S1A—C14	102.77 (15)
C16—C11—C12	118.26 (13)	C1B—S1B—C14	100.9 (3)
C16—C11—C21	121.51 (12)	S1B—C1B—H1D	109.5
C12—C11—C21	120.23 (12)	S1B—C1B—H1E	109.5
C13—C12—C11	120.98 (14)	H1D—C1B—H1E	109.5
C13—C12—H12	119.5	S1B—C1B—H1F	109.5
C11—C12—H12	119.5	H1D—C1B—H1F	109.5
C14—C13—C12	120.39 (14)	H1E—C1B—H1F	109.5
C14—C13—H13	119.8		
			
C23—N21—N22—C21	0.26 (15)	O2—C2—C32—C33	−16.9 (2)
C3—O1—C2—O2	2.32 (19)	O1—C2—C32—C33	163.70 (12)
C3—O1—C2—C32	−178.28 (11)	O2—C2—C32—C31	159.42 (13)
C5—O3—C4—O4	0.1 (2)	O1—C2—C32—C31	−19.94 (16)
C5—O3—C4—C35	−178.85 (14)	C22—C31—C32—C33	97.79 (13)
C16—C11—C12—C13	0.5 (2)	C35—C31—C32—C33	−22.52 (16)
C21—C11—C12—C13	−179.93 (13)	C22—C31—C32—C2	−78.60 (13)
C11—C12—C13—C14	−0.3 (2)	C35—C31—C32—C2	161.10 (10)
C12—C13—C14—C15	−0.5 (2)	C2—C32—C33—N31	−178.89 (11)
C12—C13—C14—S1B	−176.74 (19)	C31—C32—C33—N31	4.85 (19)
C12—C13—C14—S1A	−178.72 (14)	C2—C32—C33—C6	−1.8 (2)
C13—C14—C15—C16	1.1 (3)	C31—C32—C33—C6	−178.10 (13)
S1B—C14—C15—C16	177.66 (19)	C34—N31—C33—C32	13.4 (2)
S1A—C14—C15—C16	179.19 (15)	C34—N31—C33—C6	−164.05 (13)
C12—C11—C16—C15	0.1 (2)	C33—N31—C34—C35	−10.2 (2)
C21—C11—C16—C15	−179.48 (14)	C33—N31—C34—C7	168.98 (12)
C14—C15—C16—C11	−0.9 (3)	N31—C34—C35—C4	175.98 (11)
N21—N22—C21—C22	−0.05 (15)	C7—C34—C35—C4	−3.1 (2)
N21—N22—C21—C11	−177.88 (11)	N31—C34—C35—C31	−10.96 (18)
C16—C11—C21—N22	−49.61 (19)	C7—C34—C35—C31	169.94 (12)
C12—C11—C21—N22	130.85 (14)	O4—C4—C35—C34	16.9 (2)
C16—C11—C21—C22	132.99 (15)	O3—C4—C35—C34	−164.25 (12)
C12—C11—C21—C22	−46.5 (2)	O4—C4—C35—C31	−156.34 (14)
N22—C21—C22—C23	−0.16 (14)	O3—C4—C35—C31	22.52 (16)
C11—C21—C22—C23	177.41 (13)	C22—C31—C35—C34	−96.68 (13)
N22—C21—C22—C31	171.27 (12)	C32—C31—C35—C34	25.66 (16)
C11—C21—C22—C31	−11.2 (2)	C22—C31—C35—C4	76.59 (13)
N22—N21—C23—C22	−0.37 (15)	C32—C31—C35—C4	−161.07 (11)
C21—C22—C23—N21	0.30 (13)	C13—C14—S1A—C1A	−112.7 (3)
C31—C22—C23—N21	−171.71 (11)	C15—C14—S1A—C1A	69.2 (4)
C23—C22—C31—C32	−49.64 (15)	S1B—C14—S1A—C1A	79.4 (13)
C21—C22—C31—C32	140.55 (13)	C13—C14—S1B—C1B	−70.6 (7)
C23—C22—C31—C35	72.09 (14)	C15—C14—S1B—C1B	113.1 (7)
C21—C22—C31—C35	−97.72 (14)	S1A—C14—S1B—C1B	−57.5 (13)

### Hydrogen-bond geometry (Å, º)

**Table:**

*D*—H···*A*	*D*—H	H···*A*	*D*···*A*	*D*—H···*A*
O8—H8*A*···N22^i^	0.83 (3)	2.09 (3)	2.8982 (18)	167 (2)
O8—H8*B*···O4^ii^	0.84 (3)	2.09 (3)	2.8989 (19)	164 (2)
N21—H21···O2^iii^	0.884 (19)	1.985 (19)	2.8505 (15)	165.9 (17)
N31—H31*A*···O8^iv^	0.908 (19)	1.965 (19)	2.8561 (18)	166.6 (17)
C23—H23···S1*A*^v^	0.95	2.79	3.637 (3)	149
C31—H31···O1	1.00	2.35	2.7141 (14)	101
C31—H31···O3	1.00	2.35	2.7246 (15)	101

Symmetry codes: (i) −*x*+2, *y*−1/2, −*z*+1/2; (ii) *x*, −*y*+1/2, *z*+1/2; (iii) *x*, −*y*+3/2, *z*−1/2; (iv) −*x*+1, *y*+1/2, −*z*+1/2; (v) −*x*+2, *y*+1/2, −*z*+1/2.
